# An efficient inoculation method to evaluate virulence differentiation of field strains of sugarcane smut fungus

**DOI:** 10.3389/fmicb.2024.1355486

**Published:** 2024-04-08

**Authors:** Feng Guo, Jiaorong Meng, Ji Huang, Yanfang Yang, Shan Lu, Baoshan Chen

**Affiliations:** ^1^College of Life Science and Technology, Guangxi University, Nanning, China; ^2^State Key Laboratory for Conservation and Utilization of Subtropical Agro-Bioresources, College of Agriculture, Guangxi University, Nanning, China; ^3^Guangxi Key Laboratory of Sugarcane Biology, Ministry and Province Co-Sponsored Collaborative Innovation Center for Sugarcane and Sugar Industry, Guangxi University, Nanning, China; ^4^Academy of Sugarcane and Sugar Industry, Guangxi University, Nanning, China

**Keywords:** sugarcane, *Sporisorium scitamineum*, plantlet soaking, smut resistance, virulence differentiation

## Abstract

Sugarcane smut, caused by the fungal pathogen *Sporisorium scitamineum*, is a prominent threat to the sugarcane industry. The development of smut resistant varieties is the ultimate solution for controlling this disease, due to the lack of other efficient control methods. Artificial inoculation method is used to evaluate the virulence differentiation of pathogens. The mostly used artificial inoculation methods are soaking of the seed canes in the teliospore solution and injection of teliospores or haploid sporidia into the sugarcane sprouts. However, due to the infection nature of the pathogen that invades the sugarcane plant through meristem tissue of the sprout or shoot, the rate of successful infection is often low and fluctuated, resulting in low confidence of the assays. We recently reported a rapid and high-throughput inoculation method called plantlet soaking by using tissue culture-derived sugarcane plantlets as the test plants. Here, we compare different inoculation methods and report the characterization of parameters that may affect the sensitivity and efficiency of the plantlet soaking technique. The results showed that sugarcane plantlets were highly vulnerable to infection, even with the inoculum density at 6.0 × 10^5^ basidial spores/ml, and this method could be applied to all varieties tested. Notably, varieties showing high smut resistance in the field exhibited high susceptibility when inoculated with the plantlet soaking method, suggesting that the plantlet soaking method is a good complement to the traditional methods for screening germplasms with internal resistance. In addition, this method could also be used to monitor the variation of cellular virulence of the smut pathogen strains in the field.

## 1 Introduction

Sugarcane (*Saccharum* hybrid) is one of the most important long-duration cash crops in the world, used for sugar and biofuel production (Suprasanna et al., 2011). Sugarcane smut is a severe fungal disease first reported in Natal, South Africa, in 1877 (Luthra et al., 1940). *Sporisorium scitamineum*, the causal pathogen of sugarcane smut, is a biotrophic fungus that causes considerable yield loss and poor cane quality in almost all sugarcane producing regions of the world (Croft and Braithwaite, 2006; Bhuiyan et al., 2021). The two haploid sporidia of opposite mating types (MAT-1 and MAT-2) of the smut fungus recognize each other and fuse by conjugation to form a diploid dikaryotic hypha, which invades sugarcane and establishes a systemic infection within the apical tissue, resulting in stunting and tillering (Bhuiyan et al., 2012; Yan et al., 2016). Finally, the pathogen induces the host plant to produce a black whip-like sorus consisting of plant tissue and millions of fungal teliospores in the apex of the plant (Agisha et al., 2021; Hidayah et al., 2021). Teliospores spread to initiate infection by overcoming the bud scale barriers to colonize the meristematic tissue of the terminal or lateral buds of sugarcane plants, resulting in a new round of disease.

In-depth understanding pathogenesis of *S. scitamineum* and the molecular mechanism of pathogen-sugarcane interaction is conducive to the development of new strategies for smut control. The virulence differentiation of sugarcane smut fungus refers to its manifestation of varying pathogenic characteristics under different conditions, including its infectivity toward different sugarcane varieties, susceptibility to environmental factors, and modulation by the host’s immune response (Thushari and Costa, 2023). However, research on pathogenic mechanism of sugarcane smut fungus lags far behind *Ustilago maydis*, another smut fungus that infects maize (Martínez-Espinoza et al., 2002). Unlike the local infection of *U. maydis*, the systemic infection of *S. scitamineum* requires a longer time for the host sugarcane to show obvious disease symptoms. The lack of efficient and simple virulence detection method is one of the main reasons for the lag in the *S. scitamineum* research. Traditionally, the pathogenicity assay of *S. scitamineum* involves soaking sugarcane buds with fungal teliospores or injection of teliospores or mixed sporidia of both mating types into meristematic tissue of sugarcane sprouts derived from stalk cuttings or tissue cultures (Sun et al., 2019; Zhu et al., 2019; Cui et al., 2022). These methods have been used to characterize the virulence and physiological race differentiation of *S. scitamineum* (Deng et al., 2018) and to determine the pathogenicity of *S. scitamineum* mutant strains (Wang et al., 2019; Cai et al., 2021, 2022; Shen et al., 2022). A time frame of 60 to 180 days is required normally for the development of smut symptom and fluctuated infection rates are common with these inoculation methods.

It is challenging to breed smut resistant sugarcane varieties, given the complex genetic architecture of the sugarcane and pathogenicity differentiation of the smut fungus (Que et al., 2012; Su et al., 2016). Variation in the genetic background and planting environment of sugarcane may reduce the smut resistance of some varieties (Deng et al., 2020). Therefore, an effective and high-throughput inoculation method is needed to evaluate virulence differentiation of smut fungus. Previously, we reported a rapid and high-throughput inoculation method, called the plantlet soaking method, in which the roots of tissue culture-derived plantlets were soaked in the sporidium solution (Lu et al., 2021a). In this study, we report the characterization of parameters potentially affecting the sensitivity and efficiency of the plantlet soaking technique. We also compared the difference in infection rate and incidence rate between tissue culture-derived plantlet soaking method and traditional inoculation method. Several varieties showing high smut resistance in the field exhibited high smut susceptibility when inoculated with the plantlet soaking method, suggesting that their smut resistance do not derive from internal resistance. Moreover, differentiation in pathogenicity of six pairs of natural strains of the smut pathogen was determined.

## 2 Materials and methods

### 2.1 Plant material, fungal strains, and growth condition

Three smut susceptible sugarcane cultivars (ROC22, GT42, and LC05136) and three smut resistant cultivars (YT94128, Zhongzhe6 [ZZ6] and Zhongzhe9 [ZZ9]) were used in this study. Tissue culture-derived plantlets were used for inoculation experiments after rooting induction, as described previously (Lu et al., 2021a).

The wild-type *S. scitamineum* haploid strains, JG36, HC2, FS36, WX066, SX086 (mating type 1 [MAT-1]), and JG35, HC1, FS35, WX065, SX085 (mating type 2[MAT-2]), were isolated from different sugarcane whips in Guangxi, China. Haploid strains DH12 (MAT-1) and DH11 (MAT-2) were isolated from sugarcane whips collected from Yunnan, China. These haploid strains were cultured in liquid or on solid YEPS (1% yeast extract, 2% peptone, 2% sucrose, 1.5% agar) medium at 28°C.

### 2.2 Preparation and inoculation of tissue culture-derived plantlets

Shoot apical meristem (SAM) of healthy sugarcane plants was used to prepare callus, and the callus was induced and cultured through a series of subculturing, differentiation, and rooting steps to obtain tissue culture-derived plantlets for inoculation as described previously (Ali et al., 2008). To investigate the appropriate duration of rooting induction, tissue culture-derived plantlets with different rooting durations (20, 30, 40 and 50 days) were prepared for inoculation experiments.

The haploid sporidia of fungal strains were grown in liquid YEPS medium at 28°C, with shaking at 200 rpm. The sporidia were collected when the cell density reached OD_600_ = 1.0 by centrifugation to remove the medium. The pelleted sporidia were then resuspended in sterile water and adjusted to 6 × 10^5^ spores/ml. For inoculum preparation, MAT-1 and MAT-2 were mixed in equal volume.

The teliospores used for soaking inoculation experiments were collected from the whips of sugarcanes inoculated with JG35 × JG36. The teliospores were suspended in sterile water and the density of suspension was adjusted to 5 × 10^6^ cells/ml. The roots and bottom parts of tissue culture-derived plantlets were soaked into the haploid sporidia or teliospore suspension, and incubated at 28°C for 72 h. After inoculation, the plantlets were planted in 9-cm pots filled with nursery substrate (Guiyu, Guangxi, China), and grown in a plant growth chamber at 30°C under 12-h light (2,000 lx) /12-h dark photoperiod or in a greenhouse at 25–35°C in the summer.

### 2.3 Sprout injection inoculation

The cultivar ROC22 was grown in the field to provide healthy stalks. Sugarcane cuttings, each consisting of a single bud, were grown in a greenhouse at 30°C under 12-h light/12-h dark photoperiod for 10 days for bud germination. For injection experiments, 100 μL of sporidium suspension (6 × 10^5^ spores/ml) of JG35 × JG36 was injected into the shoot apical meristem tissue of a sprouting buds. The sprouting buds were grown in the same greenhouse. Each treatment involved three biological replicates.

### 2.4 Bud soaking inoculation

ROC22 stems, each consisting of a single lateral bud, were washed with flowing water and soaking in sporidium suspension (6 × 10^5^ spores/ml) of JG35 × JG36 at 28°C for 30 min. After inoculation, the stems were planted in nursery substrate, and grown in the same greenhouse as described preciously (Lu et al., 2021a). Each treatment involved three biological replicates.

### 2.5 Plantlet soaking inoculation

To clarify the effect of inoculum density on disease incidence rate, healthy tissue culture-derived plantlets in the same growth stage were inoculated with three different concentrations (6 × 10^5^, 6 × 10^4^, and 6 × 10^3^ spores/ml) of JG35 × JG36 inoculum. To evaluate the effect of inoculation duration on disease incidence rate, plantlets were soaked in different concentration of inoculum various time. The total number of plantlets used for each treatment was 120, with 40 plantlets per replicate.

### 2.6 Nucleic acid manipulation

The DNA of sugarcane roots and shoot apical meristems were extracted using the Plant Genomic DNA Extraction Kit (TaKaRa, Beijing, China). Polymerase chain reaction (PCR) was performed using *S. scitamineum* specific primer pair bW1-F (5′-atgtcgaccactgttctatctactc-3′), bW1-R (5′-ctaagctaggtagaaagggttggac-3′) and Taq DNA polymerase (Vazyme, Nanjing, China) for 35 cycles (95°C, 15 s; 58°C, 15 s; 72°C, 30 s).

### 2.7 Microscopy

Sugarcane tissues were sliced manually with a blade, boiled in buffer I (ethanediol: lactic acid: water = 1:1:1) for 10 min, stained with 0.4% Trypan blue for 20 s, washed three times with water, and re-boiled in buffer I for 7 min (Lu et al., 2021a). The stained tissues were visualized using the Olympus BX51 fluorescence microscope with the DP Controller software.

### 2.8 Data analysis

Data on incidence rate was recorded from the appearance of the first whip (about 30 days) until the last whip was generated. Analysis of variance for the optimization experiments were performed using version 8 of GraphPad Prism Statistics processor of One-way Analysis of Variance (ANOVA) on infection rate and incidence rate. Mean separation and comparisons were tested by Fisher’s least significance difference (LSD) test.

## 3 Results

### 3.1 Plantlet soaking protocol

**Figure 1:**
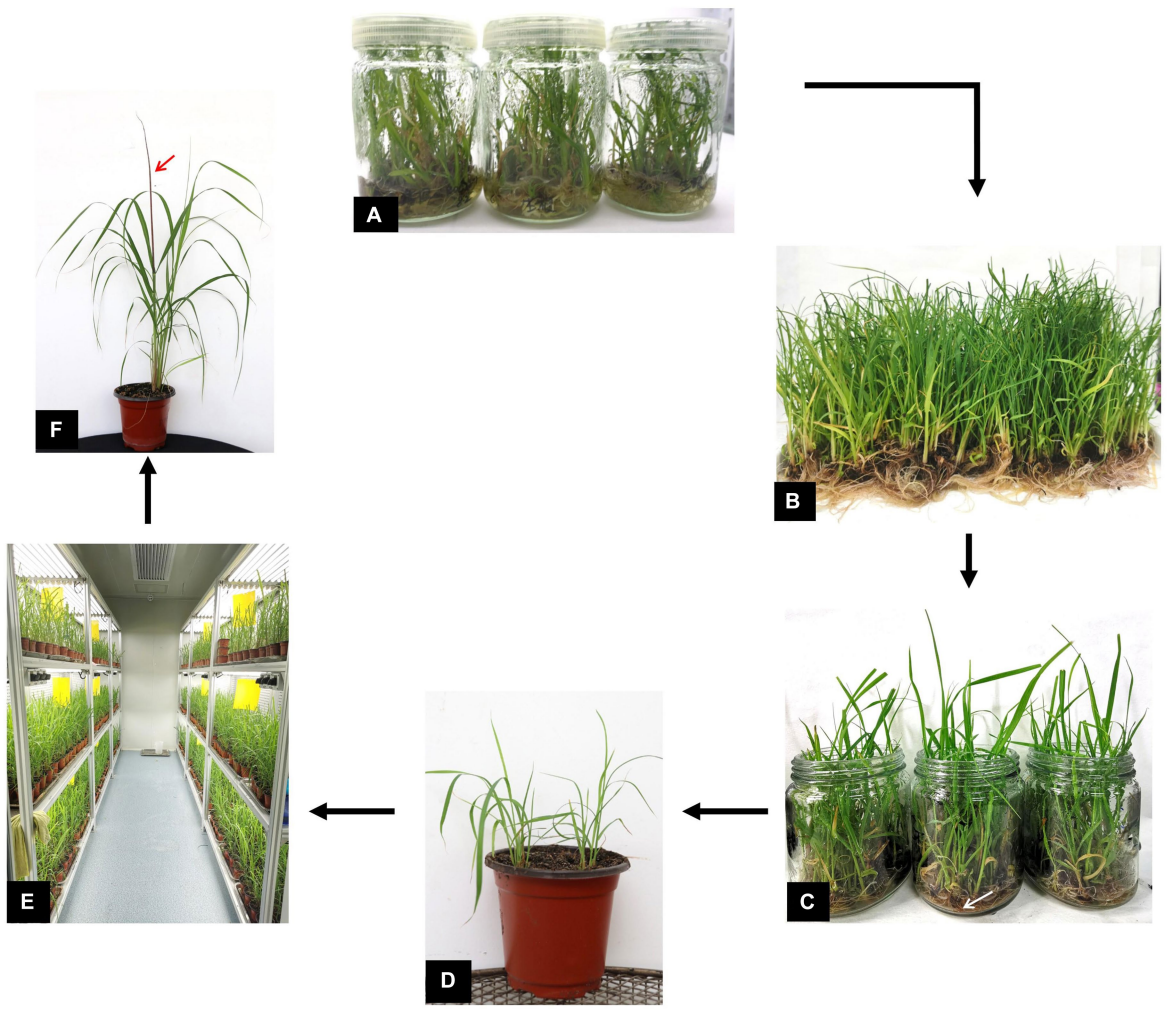
Schematic representation of the important steps involved in the plantlet soaking inoculation method. **(A)** Rooting of sugarcane tissue culture-derived plantlets for 30 days. **(B)** Clearance of seedlings with water. **(C)** Soaking of plantlets in *Sporisorium scitamineum* sporidium suspension (white arrow) in bottles. **(D)** Plantlets transplanted into small pots. **(E)** Plantlets cultured in a growth chamber. **(F)** Infected plants with black whip-like sori (red arrows).

Figure 1 illustrates the procedure of the soaking inoculation protocol. Since the survival rate of plantlets is crucial for the final pathogenicity assay, the availability of healthy plantlets is a prerequisite for the assay. To better adapt to the external environment, tissue-culture derived plantlets were exposed to air to acclimatize for 48 h before inoculation (Figure 1A). Culture medium attached to the roots of the plantlets was removed by washing with water to facilitate the attachment of fungal inoculum (Figures 1B,C). After incubation with the pathogen at 28°C for 3 days, the plantlets were transferred to small pots containing nursery substrate (Figure 1D). Since the plantlets were small in size, they could easily be kept in a regular growth chamber (Figure 1E). Whip-like sori, the hallmark symptom of sugarcane smut, usually appeared at approximately 35 days in average (Figure 1F).

### 3.2 Comparative analysis of different inoculation methods

To compare the effects of different inoculation methods on infection rates, the ROC22 germinating bud/soaking, sprouting bud/injection, and tissue culture plantlet/soaking were inoculated with sporidia (JG35 × JG36) and the infection was verified by histopathological analysis. At all of the three time points (10, 20, and 30 days post inoculation), plantlet soaking yielded much higher infection rates, 66.7, 65 and 93.3%, versus 5, 0 and 20% for the bud soaking, and 0, 10 and 60% for sprout injection (Table 1). The whip rates of bud soaking (23.2%) and sprouting injection (70.8%) were significantly lower than that the tissue culture plantlet soaking (93.7%). Of significance, the time required for whip emergence by plantlet soaking was the shortest among the three inoculation methods (Table 1).

**Table 1:** Comparison of inoculation methods using sporidium as inoculum.

Method	Plant material	Infection rate (%)*			Whip emergence post-inoculation (d)**	Whip rate (%)***
		10 dpi	20 dpi	30 dpi		
Soaking	Germinating bud	1/20 (5)^bB^	0/20 (0)^cC^	4/20 (20)^cA^	60–116^a^	20/86 (23.2)^c^
Injection	Sprouting bud	0/20 (0)^bC^	2/20 (10)^bB^	12/20 (60)^bA^	30–100^b^	68/96 (70.8)^b^
Soaking	Tissue culture plantlet	16/24 (66.7)^aB^	26/40 (65)^aB^	28/30 (93.3)^aA^	30–60^c^	89/95 (93.7)^a^

*Data represents the sum of plantlets with hyphae/ total number of plantlets (infection rate, %). The infection rates were derived from three replicated experiments. Different lowercase letters represent significant differences within the same column, uppercase letters represent significant differences within the same row, as determined using ANOVA and Fisher’s LSD test (*p* < 0.05).

**Time range indicates the day of the appearance of the first whip to the day of appearance of the last whip. Different lowercase letters represent significant differences within the same column, as determined using ANOVA and Fisher’s LSD test (*p* < 0.05).

***Data represents the number of plantlets with whip/ total number of surviving plants (whip rate, %). The plantlets were monitored for up to 120 days post-inoculation. The whip rates were derived from three replicated experiments. Different lowercase letters represent significant differences within the same column, as determined using ANOVA and Fisher’s LSD test (*p* < 0.05).

Three other inoculation methods (teliospores soaking, sporidia soaking and sporidia injection) were compared under the same conditions using tissue culture-derived plantlets as testing material (Supplementary Figure S1). The results showed that the whip rates of teliospores soaking (52.0%) and sporidia injection (33.3%) were significantly lower than that the sporidium soaking (90.7%). Interestingly, the time for plantlets inoculated with sporidia soaking to produce whips was also significantly shorter than that of plantlets inoculated with the other two methods (Table 2).

**Table 2:** Effect of inoculation methods on smut rate on tissue culture plantlets.

Method	Whip emergence post-inoculation (d)*	Infection rate (%)**	Whip rate (%)***
Sporidium soaking	30–80^c^	103/108 (95.4)^a^	98/108 (90.7)^a^
Teliospore soaking	50–100^b^	79/127 (62.2)^c^	66/127 (52.0)^b^
Sporidium injection	70–100^a^	60/78 (76.9)^b^	26/78 (33.3)^c^

*Time range indicates the day of the appearance of the first whip to the day of appearance of the last whip. Different lowercase letters represent significant differences within the same column, as determined using ANOVA and Fisher’s LSD test (*p* < 0.05).

**Data represents the sum of plantlets with hyphae/ total number of surviving plantlets (infection rate, %). The infection rates were derived from three replicated experiments. Different lowercase letters represent significant differences within the same column, as determined using ANOVA and Fisher’s LSD test (*p* < 0.05).

***Data represents the number of plantlets with whip/ total number of surviving plants (whip rate, %). The plantlets were monitored for up to 100 days post-inoculation. The whip rates were derived from three replicated experiments. Different lowercase letters represent significant differences within the same column, as determined using ANOVA and Fisher’s LSD test (*p* < 0.05).

### 3.3 Optimization of soaking inoculation conditions

**Figure 2:**
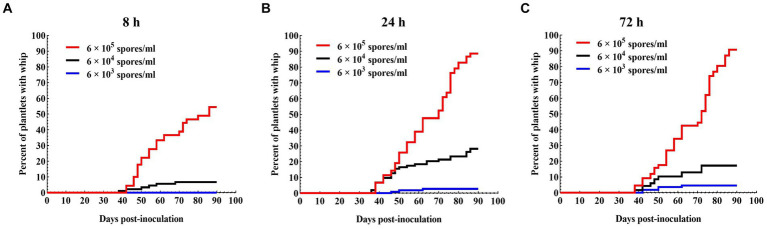
Progression of whip development induced by a mixed solution of JG35 × JG36 sporidium suspension with different inoculation durations. **(A)** 8 h; **(B)** 24 h; **(C)** 72 h. Plantlets were kept in a growth chamber.

To optimize the inoculation parameters, inoculum concentration and plantlet-pathogen incubation time were investigated. Incubation time of 8 h yielded lowest survival rates (74.2–80%) and whip rates (0–52.2%), but no significant difference was found between 24 h (survival rates 85.8–90.8%; whip rates 2.8–88.6%) and 72 h (survival rates 90–96.7%; whip rates 4.5–90.7%). On the other hand, inoculum concentration did not seem to have a significant impact on the survival rate of the plantlets, but had a great impact on whip rate, from 90.7% at 6.0 × 10^5^ spores/ml to 4.5% at 6.0 × 10^3^ spores/ml for the same incubation time of 72 h (Table 3; Figure 2). Taken together, the sporidium concentration at 6 × 10^5^ spores/ml, and inoculation duration of 72 h were the best combination for inoculation.

**Table 3:** Effect of incubation time and inoculum density on the survival rate and whip rate of sugarcane inoculated with *Sporisorium scitamineum* strains JG35 and JG36.

Duration of inoculation	Survival rate (%)*			Whip rate (%)**		
	6.0 × 10^5^ spores/ml	6.0 × 10^4^ spores/ml	6.0 × 10^3^ spores/ml	6.0 × 10^5^ spores/ml	6.0 × 10^4^ spores/ml	6.0 × 10^3^ spores/ml
8 h	90/120 (75)^bA^	89/120 (74.2)^cA^	96/120 (80)^bA^	49/90 (52.2)^cA^	6/89 (6.7)^cB^	0/96 (0)^cC^
24 h	105/120 (87.5)^aA^	103/120 (85.8)^bA^	109/120 (90.8)^aA^	93/105 (88.6)^bA^	29/103 (28.2)^aB^	3/109 (2.8)^bC^
72 h	108/120 (90)^aA^	116/120 (96.7)^aA^	110/120 (92.1)^aA^	98/108 (90.7)^aA^	20/116 (17.2)^bB^	5/110 (4.5)^aC^

* Data represents the number of surviving plantlets/ total number of inoculated plantlets (survival rate, %) with three different inoculum densities (6.0 × 10^5^, 6.0 × 10^4^, 6.0 × 10^3^ spores/ml). The roots of plantlets were soaked for different durations (8, 24, and 72 h) in the sporidium suspension, which was prepared by mixing equal volumes (10 mL) of suspension cultures of JG35 and JG36. Different lowercase letters represent significant differences within the same column, uppercase letters represent significant differences within the same row, as determined using ANOVA and Fisher’s LSD test (*p* < 0.05).

** Data represents the number of plants with whip/ total number of surviving plants (whip rate, %). The plants were monitored for up to 90 days post-inoculation, and the symptoms were recorded. The survival rate and whip rates were derived from three replicated experiments. Different lowercase letters represent significant differences within the same column, uppercase letters represent significant differences within the same row, as determined using ANOVA and Fisher’s LSD test (*p* < 0.05).

Rooting duration (root induction of tissue culture-derived plantlet) might also influence the survival rate and whip rate. Inoculation of plantlets with 20 to 50 day-rooting duration yielded different whip rates, with the highest of 88.9% for the 30 day rooting and the lowest of 58.7% for the 50 day rooting (Table 4).

**Table 4:** Effect of the plantlet rooting duration on the whip rate.

Rooting time (d)*	No. of plants with whip/No. of plants inoculated (%)**
20	45/52(86.5)^a^
30	64/72 (88.9)^a^
40	45/63 (71.4)^b^
50	44/75 (58.7)^c^

*Rooting time: the number of days for which the tissue culture-derived plantlets were allowed to grow on the rooting medium before inoculation with the pathogen. The density of inoculum was set at 6.0 × 10^5^ spores/ml, and the plants were monitored for up to 90 days post-inoculation.

**Data represents the number of plantlets with whip/ total number of inoculating plants (whip rate, %). The whip rates were derived from three replicated experiments. Different letters represent significantly different using Fisher’s LSD test (*p*<0.05).

### 3.4 Infection route of *Sporisorium scitamineum* in tissue culture-derived plantlets

**Figure 3:**
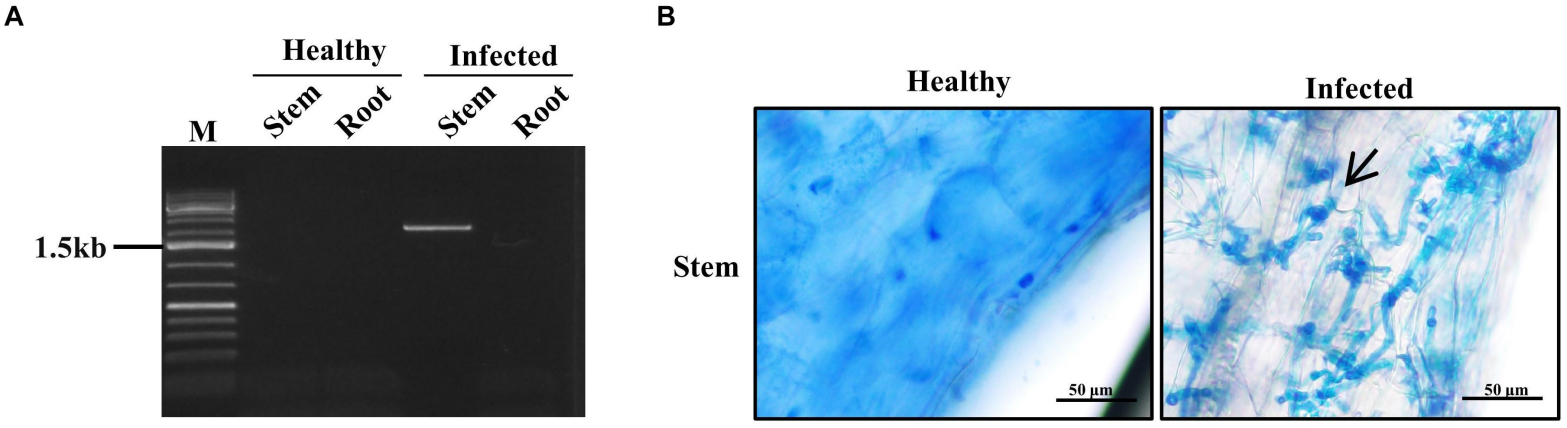
Localization of smut fungus in the infected sugarcane plantlets. Samples were collected 9 days post inoculation. **(A)** PCR of the sugarcane plant tissues. Stem represents stem meristem tissue and Root represents root meristem tissue. **(B)** Microscopy of sugarcane stem meristem tissues. Arrow indicates the fungal mycelium. Bar = 50 μm.

In order to investigate the location of the pathogen during the infection process, we examined the root meristem and the stem meristem tissues using PCR and histology at day 9 post inoculation. PCR analysis with primer sets specific to *bW1* gene (2,151 bp) of the *S. scitamineum* showed that the positive band was only detected from the stem meristem tissue, but not the root meristem tissue (Figure 3A). Histological analysis showed that fungal mycelia were present in the stem meristem tissue (Figure 3B). These results suggest that the pathogen only invades the stem apical meristem tissue but not the root meristem tissue.

### 3.5 Identification of external resistance in sugarcane

**Figure 4:**
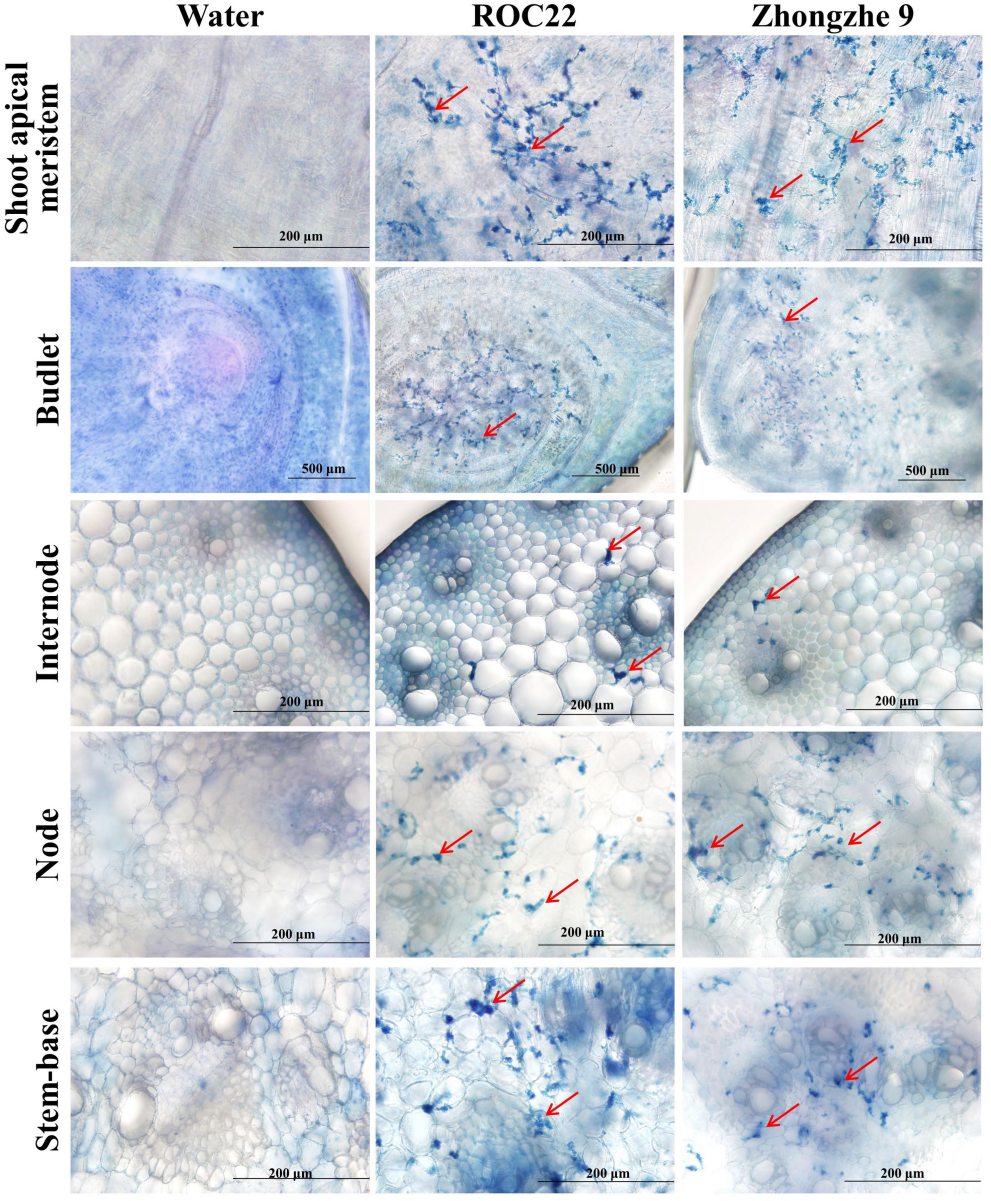
Visualization of hyphae in the tissues of infected plants. Histopathological analysis was performed by dissecting the tissues of the stalk. The sections were stained with 0.4% trypan blue. Red arrows indicate hyphae. No hypha was detected in plantlets inoculated with sterile water. Bar = 200 μm.

Field resistance could derive from either physical resistance (external resistance, i.e., resistance against penetration) or internal resistance (cell immunity), or both types. In the field, varieties ROC22, GT42, and LC05136 were smut sensitive, whereas YT94128, ZZ6, and ZZ9 were smut resistant. Among these varieties, ZZ6 and ZZ9 had been growing in different sugarcane plantations in China for 3–6 years. Field surveys revealed that smut rate was 0% in the new plantations of both these varieties, and up to 0.53% in the ratoon-derived crop over three consecutive years, demonstrating that both varieties are highly resistant to the smut. However, the whip rates of both these resistant varieties were 77.3–90.9%, indistinguishable from the susceptible varieties (*P* > 0.05), when they were inoculated with the plantlet soaking method (Table 5; Supplementary Figure S2). Histopathological analysis revealed that mycelia were mainly present in the stem meristem tissue, nodes, and buds, with a small amount distributed in the internodes (Figure 4; Supplementary Figure S3). These results indicate that the field resistance of YT94128, ZZ6, and ZZ9 are mainly due to external resistance, instead of internal resistance.

**Table 5:** Effect of variety on the infection rate infected with JG35 × JG36 strains*.

Sugarcane variety	Field resistance^a^	Whip emergence post-inoculation (d)^b^	No. of plants with whip/No. of plants inoculated (%)^c^
ROC22	HS	35–70	29/39 (74.4)
		35–68	28/33 (84.8)
GT42	HS	40–70	23/32 (71.9)
		40–70	26/29 (89.7)
LC05136	HS	35–70	34/40 (85.0)
		34–78	27/35 (77.1)
YT94128	HR	33–70	31/35 (88.6)
		30–70	34/35 (97.1)
Zhongzhe6	HR	30–65	16/18 (88.9)
		30–70	10/11 (90.9)
Zhongzhe9	HR	30–65	17/22 (77.3)
		33–70	16/19 (84.2)

*Plantlets were kept in a greenhouse.

^a^HS: highly susceptible; HR: highly resistant.

^b^Time range indicates the day of the appearance of the first whip to the day of appearance of the last whip. There was no significant difference in the time range of black whip among different sugarcane varieties (*p*>0.05).

^c^Whip rates were derived from two replicated experiments. There was no significant difference in whip rate among different sugarcane varieties (*p*>0.05).

### 3.6 Evaluation of virulence differentiation of the field-isolated fungal strains

**Figure 5:**
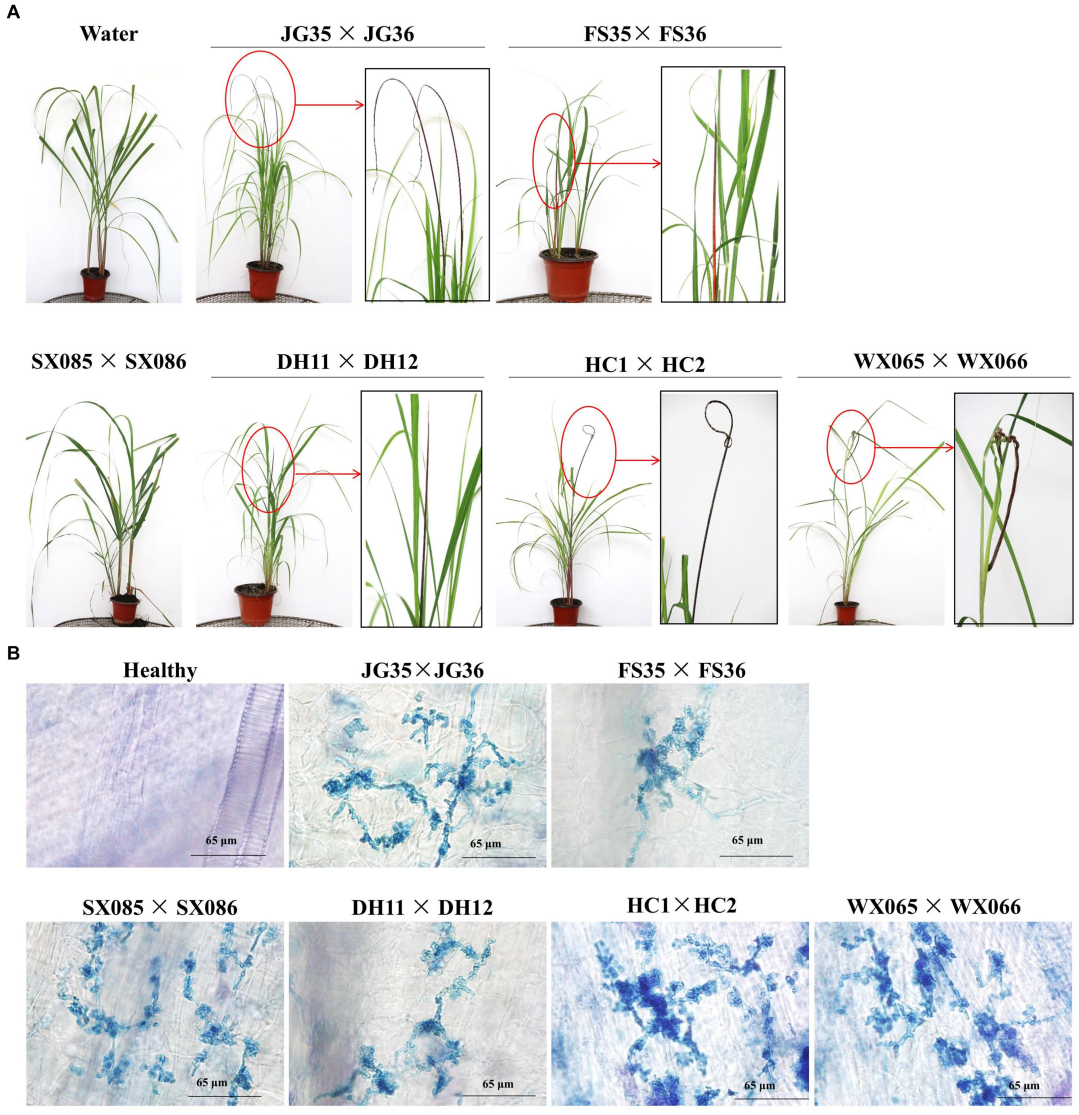
Pathogenicity assay of field strains of the smut fungus. **(A)** Black whips incited by different strains. Boxed images are the enlarged portions of the whips. No whip was formed in plants infected with SX085 × SX086. **(B)** Histopathology of the shoot apical meristem tissues of sugarcane plantlets. Mycelia were present in the plants infected with all strains, including SX085 × SX086.

We next wondered if the plantlet soaking method could be used to distinguish the virulence variation among the pathogen strains. Six pairs of *S. scitamineum* haploid strains isolated from different sugarcane varieties and planting areas were used to inoculate the tissue culture plantlets derived from the smut-susceptible sugarcane variety ROC22. Whip rates of 86.8, 95.3, and 96.6% were recorded for DH11 × DH12, FS35 × FS36, and JG35 × JG36, while 2.3 and 4.7% whip rates for WS065 × WS066 and HC1 × HC2, and 0% whip rate for SX085 × SX086, in the observation period of 100 days (Table 6). Histopathological examination revealed that the majority of the inoculated plantlets were infected by HC1 × HC2 (81 out of 85), while only a fraction of the inoculated plantlets were infected by WS065 × WS066 (12 out of 86) and SX085 × SX086 (2 out of 109) (Figure 5; Table 6). The results indicate that there was a significant degree of virulence differentiation in the population of the smut fungus in the field.

**Table 6:** Virulence assay of field-isolated strains of *Sporisorium scitamineum.*

Inoculum	Whip emergence post-inoculation (d)*	Infection rate (%)**	Whip rate (%)***
JG35 × JG36	30–80^d^	86/87 (98.9)^a^	84/87 (96.6)^a^
FS35 × FS36	37–100^c^	83/86 (96.5)^a^	82/86 (95.3)^a^
DH11 × DH12	35–96^c^	62/68 (91.2)^a^	59/68 (86.8)^b^
SX085 × SX086	–	2/109 (1.8)^c^	0/109 (0)^e^
HC1 × HC2	90–110^a^	81/85 (95.3)^a^	4/85 (4.7)^c^
WX065 × WX066	80–100^b^	12/86 (14.0)^b^	2/86 (2.3)^d^
Water	–	0/40 (0)^d^	0/40 (0)^e^

*Time range indicates the day of the appearance of the first whip to the day of appearance of the last whip. Different lowercase letters represent significant differences within the same column, as determined using ANOVA and Fisher’s LSD test (*p* < 0.05).

**Data represents the sum of plants with whips and plants with hyphae/ total number of surviving plants (infection rate, %). The infection rates were derived from three replicated experiments. Different lowercase letters represent significant differences within the same column, as determined using ANOVA and Fisher’s LSD test (*p* < 0.05).

***Data represents the number of plants with whip/ total number of surviving plants (whip rate, %). The plants were monitored for up to 110 days post-inoculation. The whip rates were derived from three replicated experiments. Different lowercase letters represent significant differences within the same column, as determined using ANOVA and Fisher’s LSD test (*p* < 0.05).

## 4 Discussion

As a biotrophic pathogen, *S. scitamineum* has evolved an ability to colonize within the apical meristem tissue of the host sugarcane in a persistent manner, without apparent attack by the immune system of the host plant (Rajput et al., 2022). This latent infection nature makes it difficult to observe early symptom develoment and leads to an extended time for eveluation of crop resistance or pathogen pathogenicity. Thus, a rapid and reliable inoculation method would facilitate the investigation of smut pathogen–sugarcane interaction (Rajput et al., 2019). In this regard, the soaking inoculation method offers a solusion with several merits. Firstly, using sporidia as inoculum could accelerate symptom develoment, likely by avoiding the process of teliospore germination (Table 2). Secondly, high infection rate of this method (Table 1) helps to solve the problem of drastic fluctuation in whip rate between experiments or replicates, as oftern encountered by using the traditional methods. In addition, high infection rate is also helpful in quantifying the virulence variation among pathogen strains or degree of resistance of sugarcane varieties. Thirdly, tissue culture procedures make it easier to obtain uniform plant materials in large quantities in all seasons. This is particularly important for high-throughput screening of virulence variation in pathogen population (Table 6) or a large number of mutants generated in the functional genomics of the fungus. In fact, this method has been used to determine the pathogenicity of *S. scitamineum* mutants generated by T-DNA insertion or gene-targeting deletion (Lu et al., 2021a,b, 2022; Zhang et al., 2022).

In agricultural practice, sugarcane is planted using the mature stalks as seeds (Jain et al., 2010). Sprouts emerged from the buds of a stalk cutting are surrounded by bud scales, which are assumed to protect the shoot apical meristem from pathogen infection, the structural basis of external resistance to sugarcane smut (Waller, 1970; Marques et al., 2018; Vicente et al., 2021). Chemical metabolites, such as polyphenols, secreted by the bud scales may also contribute to the external resistance (Waller, 1969; Dean, 1982). Recently, Liu et al. reported that susceptible varieties had higher levels of hexacosanol and octacosanol on the surface of sugarcane buds and these chemical compounds promoted the germination of *S. scitamineum* teliospores *in vitro* (Liu et al., 2022). Once the external defense barrier is broken, the smut fungus penetrates into the shoot apical meristem, where the internal resistance response is activated, either by pathogen-associated molecular pattern-triggered immunity (PTI), and/or by effector-triggered immunity (ETI) (Peng et al., 2018; Ramirez-Prado et al., 2018). Unlike bud sprouts, tissue culture-derived plantlets are loose at the base and are not protected by bud scales. We reason that this loose structure in tissue culture-derived plantlets facilitates infection by providing the smut pathogen easy access to the shoot apical meristem, thus resulting in extremely high infection rate. Therefore, the soaking inoculation method is useful to identify external resistance in combination with field evaluation. In this regard, smut resistance of YT94128, ZZ6, and ZZ9 were concluded to stem mainly from the external resistance. Equally importance is that this soaking method could also be used to screen sugarcane germplasm with internal resistance.

## 5 Conclusion

In summary, we report a rapid and reliable plantlet soaking method for inoculation of sugarcane with *S. scitamineum*, and the characterization of parameters that may affect the sensitivity and efficiency of this technique in sugarcane. By using this method, virulence differentiation of six field-isolated strains from Southern China was determined and three varieties showing high smut resistance in the field was classified as external resistance.

## Data availability statement

The original contributions presented in the study are included in the article/Supplementary material, further inquiries can be directed to the corresponding author.

## Author contributions

FG: Investigation, Methodology, Writing – original draft. JM: Methodology, Writing – review & editing. JH: Investigation, Writing – review & editing. YY: Investigation, Writing – review & editing. SL: Funding acquisition, Investigation, Supervision, Writing – original draft, Writing – review & editing. BC: Funding acquisition, Project administration, Supervision, Writing – original draft, Writing – review & editing.
