# Supplementary material for: An efficient inoculation method to evaluate virulence differentiation of field strains of sugarcane smut fungus
Source: Front Microbiol. 2024 Apr 8;15:1355486. doi: 10.3389/fmicb.2024.1355486 (PMC11033459; doi:10.3389/fmicb.2024.1355486)
Supplement: Supplementary file 1 [file Data_Sheet_1_2.pdf]

Supplementary Material

An efficient inoculation method to evaluate virulence differentiation of field strains of sugarcane smut fungus

Feng Guo[1], Jiaorong Meng[2,3], Ji Huang[2,4], Yanfang Yang[2], Shan Lu[2,3,4*], Baoshan Chen[2,3,4*]

*** Correspondence:** Shan Lu: lushan@gxu.edu.cn, Baoshan Chen: chenyaoj@gxu.edu.cn

Supplementary Figures

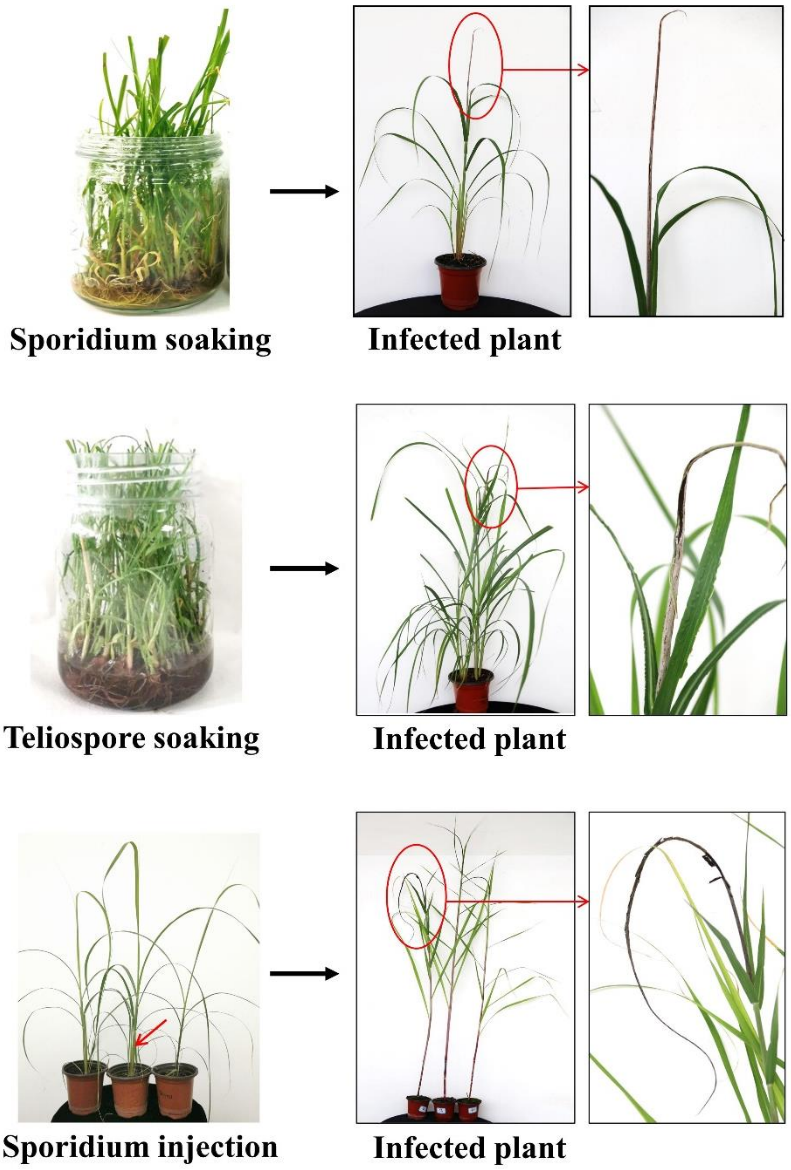

Supplementary Figure S1. Disease symptoms in tissue culture-derived plantlets of haploid sporidia soaking, teliospores soaking and sporidia injection inoculation. Boxed images are the enlarged portions of the whips.

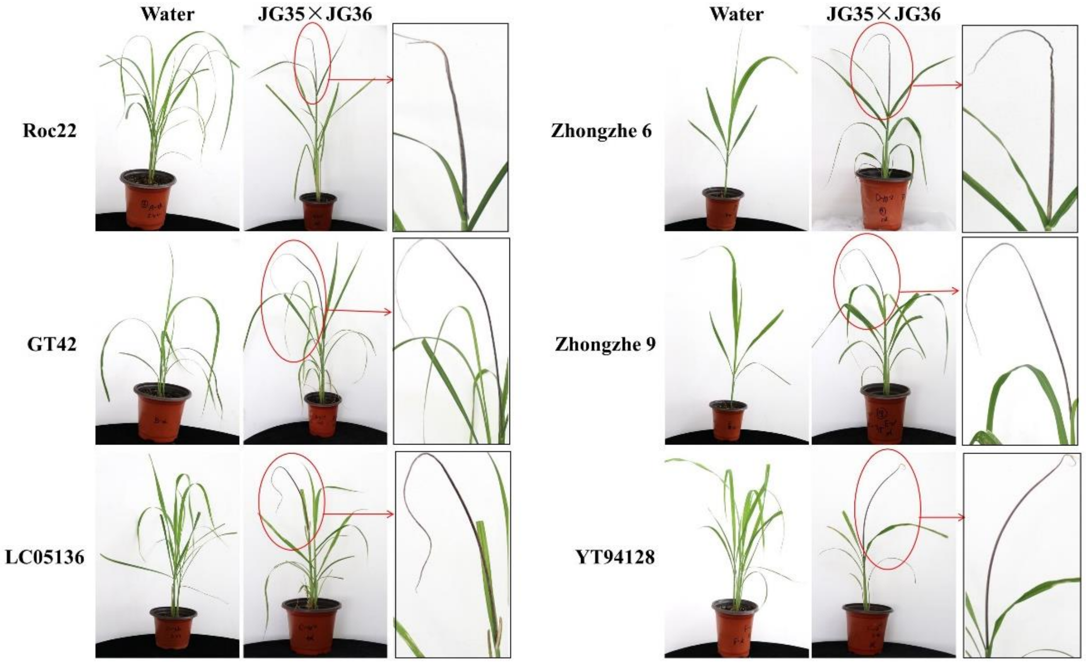

Supplementary Figure S2. Black whip incited in plantlet of six varieties. Boxed images are the enlarged portions of the whips.

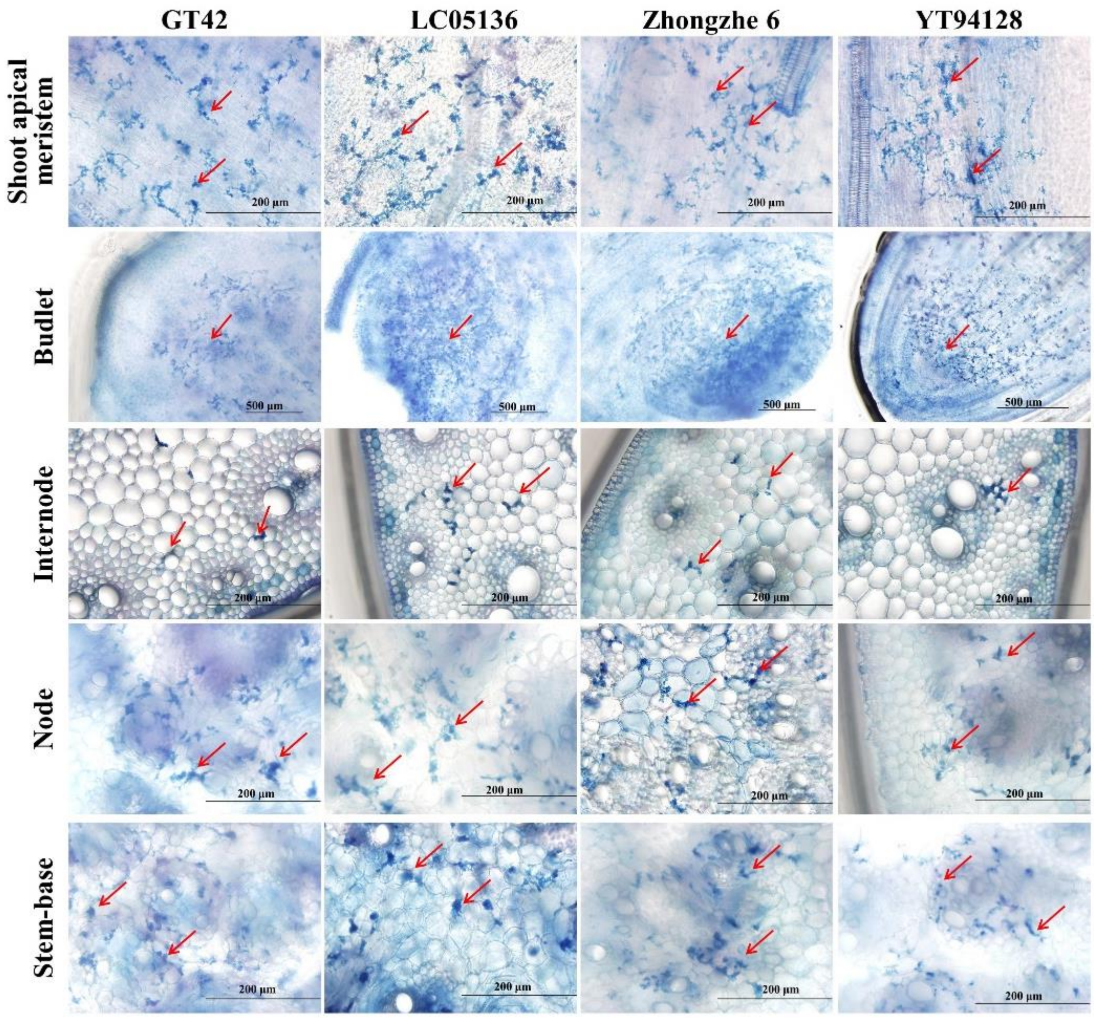

Supplementary Figure S3. Visualization of hyphae in the tissues of infected plants. Histopathological analysis was performed by dissecting the tissues of the stalk. The sections were stained with 0.4% trypan blue. Red arrows indicate hyphae. Bar = 200 μm.
